# Targeting hypoxia-inducible factors for breast cancer therapy: A narrative review

**DOI:** 10.3389/fphar.2022.1064661

**Published:** 2022-12-01

**Authors:** Shuang Luo, Yu Jiang, Anfu Zheng, Yueshui Zhao, Xu Wu, Mingxing Li, Fukuan Du, Yu Chen, Shuai Deng, Meijuan Chen, Wanping Li, Xiaobing Li, Li Gu, Yuhong Sun, Zhangang Xiao, Jing Shen

**Affiliations:** ^1^ Laboratory of Molecular Pharmacology, Department of Pharmacology, School of Pharmacy, Southwest Medical University, Luzhou, China; ^2^ Cell Therapy and Cell Drugs of Luzhou Key Laboratory, Luzhou, China; ^3^ South Sichuan Institute of Translational Medicine, Luzhou, China; ^4^ Department of Pharmacy, The Second People’s Hospital of Jiangyou, Mianyang, China; ^5^ Department of Pharmacy, The People’s Hospital of Wusheng, Guang’an, China; ^6^ Department of Oncology, The Affiliated Hospital of Southwest Medical University, Luzhou, China

**Keywords:** hypoxia-inducible factors, breast cancer, drug delivery systems, tumor microenvironment, angiogenesis, glycolysis

## Abstract

Hypoxia-inducible factors (HIFs), central regulators for cells to adapt to low cellular oxygen levels, are often overexpressed and activated in breast cancer. HIFs modulate the primary transcriptional response of downstream pathways and target genes in response to hypoxia, including glycolysis, angiogenesis and metastasis. They can promote the development of breast cancer and are associated with poor prognosis of breast cancer patients by regulating cancer processes closely related to tumor invasion, metastasis and drug resistance. Thus, specific targeting of HIFs may improve the efficiency of cancer therapy. In this review, we summarize the advances in HIF-related molecular mechanisms and clinical and preclinical studies of drugs targeting HIFs in breast cancer. Given the rapid progression in this field and nanotechnology, drug delivery systems (DDSs) for HIF targeting are increasingly being developed. Therefore, we highlight the HIF related DDS, including liposomes, polymers, metal-based or carbon-based nanoparticles.

## Graphical Abstract

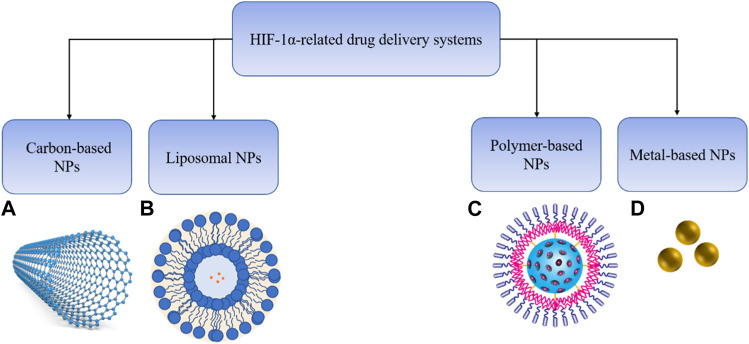

## 1 Introduction

Breast cancer (BC) is one of the major diseases affecting women’s health and the leading cause of female death worldwide. According to the Global Cancer Statistics 2020 report, in terms of morbidity, the number of new cases of BC in 2020 reached 2.3 million, accounting for about 11.7% of the total cases (Sung et al., 2021). Triple-negative breast cancer (TNBC) is defined as BC lacking expression of estrogen (ER), progesterone (PR) and human epidermal growth factor receptor 2 (HER2) and is classified as one of basal-like BC (BLBC) (Wolff et al., 2013). BC treatment is divided into systemic and localized treatment based on BC subtype and degree of metastasis. For non-metastatic BC, local therapy is mainly used to eradicate the tumor through surgical resection and radiotherapy, while for metastatic BC or more aggressive triple-negative BC, systemic therapy consisting of chemotherapy and immunotherapy are used to prevent tumor metastasis and recurrence (Waks and Winer, 2019). Although surgery, radiotherapy, chemotherapy, targeted therapy, and immunotherapy have improved the survival and quality of life of BC patients in recent decades, the mortality rate of BC is high due to lack of therapeutic targets and chemotherapy resistance. Therefore, finding effective therapeutic targets and reducing drug resistance are indispensable in the treatment of BC (Veronesi et al., 2005; Barzaman et al., 2020).

The rapid proliferation of the tumor beyond its surrounding vasculature results in the normal oxygen level to drop to less than 2%, and the area with low oxygen is called the hypoxic area. Hypoxia promotes tumor plasticity and heterogeneity and a more aggressive and metastatic phenotype, which is seen in many solid tumors and is an important feature of the BC tumor microenvironment (Harris, 2002). Hypoxia-inducible factor (HIF) is a key marker of hypoxia and a core player involved in cell adaptation to hypoxia (Huang et al., 2017; Rani et al., 2022). Recently, nanoparticles (NP), as an effective drug delivery method have attracted special interest for cancer treatment. Various ongoing studies aim to optimize this method to ultimately reduce adverse reactions caused by traditional methods. So far, the NP used in drug delivery research for targeting HIF in BC includes liposomes NPs, polymers NPs, metal-based NPs or carbon-based NPs. Using NP for drug delivery has many advantages: 1) It improves the problems related to poor drug solubility and bioavailability; 2) It enhances the permeability of targeted drugs to cancer cells and slowly releases drugs; 3) NPs are very small (1–100 nm), non-toxic, biodegradable, and cancer drugs can be easily loaded onto these particles; 4) Delivery of multiple drugs with differing properties can be achieved (Farokhzad and Langer, 2009; Burgess et al., 2010). Compared with standard chemotherapy methods, nano carriers can significantly reduce the damage to healthy cells and tissues. Therefore, nano carriers may be used in clinical applications in the future in NP based drug delivery system (DDS) or in combination therapy. The main purpose of this review is to briefly summarize the mechanism of HIF-1 mediated angiogenesis, glycolysis, metastasis and drug resistance. Furthermore, we discuss the current therapeutic strategies targeting HIF-1, including HIF-1α inhibitors in preclinical and clinical studies, as well as small molecules targeting HIF-1α related signaling pathway. In addition, we emphasize the current progress in HIF related drug delivery systems.

## 2 Materials and methods

### 2.2 Data and processing

The expression profile and related clinical follow-up information of BRCA were download from The Cancer Genome Consortium (TCGA) database. A total of 1098 tumor samples and 113 normal samples were included.

### 2.2 Expression analysis

The expression of HIF1A in different subgroups was matched by clinical annotation information. All statistical analyses were implemented by R (v4.1.3) language. Statistical differences between each two subgroups were calculated by Wilcoxon test.

### 2.3 Literature search

We mainly used “NCBI-Pubmed” to conduct online literature search of all articles published in English over the past 10 years. The search words include “hypoxia and HIF”, “HIF and breast cancer”, “HIF and angiogenesis and breast cancer”, “HIF inhibitor and breast cancer”, sorted by “best match”. Search results were selected by year ranging from the most recent to the earlier ones and also by impact factor of the article. Clinical studies were searched by key words “HIF and breast cancer” and article type set as clinical trials in Pubmed and also searched in ClinicalTrials.gov.

## 3 Hypoxia-inducible factors

**FIGURE 1:**
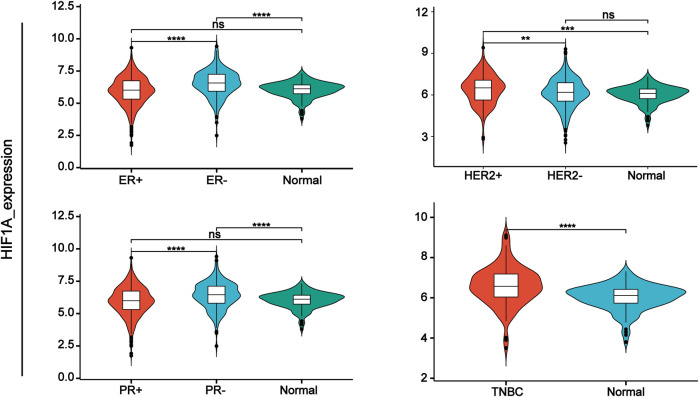
HIF-1α expression in different types of breast cancer tissue samples compared with normal tissue from TCGA database.

**FIGURE 2:**
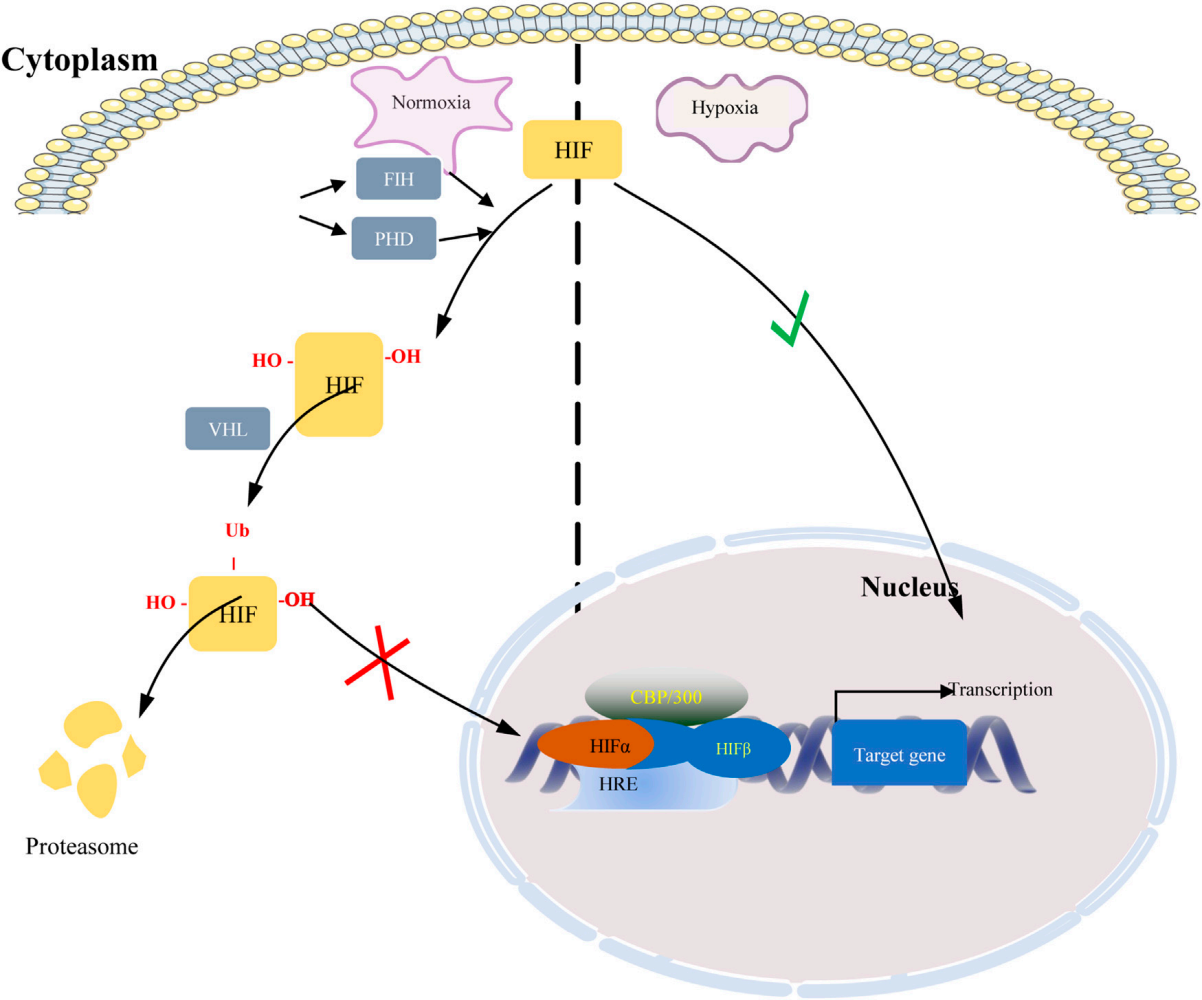
Regulation of HIF under normoxic and hypoxic conditions. When oxygen is abundant, HIF is hydroxylated by prolyl hydroxylase domain protein (PHD) enzymes at two specific proline residues, enabling it to bind VHL. VHL targets hydroxylated HIF subunits for ubiquitin-mediated proteasomal degradation. Under hypoxic conditions, inactivation of PHD and FIH-1 results in HIF stabilization and translocation into the nucleus where it stabilizes and dimerizes with HIF-1β, which together with the co-transcription factors p300 and CBP, drives hypoxia Transcription of target genes of response elements (HREs).

Hypoxia-inducible factors (HIFs) are transcription factor responsible for activation of hypoxia genes. They are heterodimers belonging to the basic helix-loop-helix/Per-Arnt-Sim (bHLH/PAS) transcription factors, which are composed of an oxygen-regulated 120 kDa α subunit, and an oxygen-independent 91–94 kDa β subunit (Loboda et al., 2010). Three HIF-α subtypes (HIF-1α, HIF-2α and HIF-3α) have been reported, HIF-1α is the most classical and widely studied (Konisti et al., 2012). HIF-1α and HIF-2α share 48% amino acid sequence homology and similar domain arrangement, while there are different hypoxia-sensitivities for different prolyl hydroxylase sites (Pro564 and Pro402 in HIF-1α, Pro405 and Pro531 in HIF-2α) (Iyer et al., 1998; Jokilehto and Jaakkola, 2010). HIF-1α is thought to be a key coordinator of cancer cell responses to the hypoxic microenvironment by regulating metabolic reprogramming, angiogenesis, stem cell maintenance, matrix remodeling, metastasis and resistance to chemoradiotherapy (Schito and Semenza, 2016). Numerous studies have shown that the expression of HIF-1α was elevated in BC and high expression of HIF-1α predicts poor patient survival (Talks et al., 2000; Rajkovic-Molek et al., 2014; Cui and Jiang, 2019; Shamis et al., 2021). By analyzing TCGA data, we found that HIF-1α was highly expressed in TNBC. Moreover, its expression was higher in ER- and PR-compared with ER+ and PR + BC respectively (Figure 1). HIF-2α may play an important role in a variety of cells other than endothelial cells as well as in tumorigenesis Hu et al., 2003). HIF-3α mainly depends on other HIF complexes (Bristow and Hill, 2008). Under normal microenvironment, the HIFα-subunit is degraded with the aid of E3 ligase through hydroxylation by prolyl hydroxylase domain protein (PHD) and polyubiquitination of VonHippel-Lindau (VHL). Factor-inhibiting HIF-1α (FIH-1) is another transcriptional regulator of HIF-1α and HIF-2α, which interferes the binding of HIF to co-transcription factors (Rani et al., 2022). Under hypoxic conditions, PHD activity is reduced. So, HIF-α in cytoplasmic is accumulated and translocated to the nucleus, where α subunit dimerizes with β-subunit and induces transcription of target genes by binding to hypoxia response elements (HREs) in promoters (Figure 2) (Wang et al., 1995; Semenza, 2014; de Heer et al., 2020). Recently, it has been described that, HIF-1α and HIF-2α can be regulated in an oxygen-independent by regulators such as hypoxia-associated factor (HAF), small ubiquitin-related modifier (SUMO)-specific protease 1 (SENP1), and Int6/eukaryotic initiation factor (eIF) 3e (Hashimoto and Shibasaki, 2015). Enhanced expression of HIF-targeted genes is associated with many human diseases, including ischemic cardiovascular disease, stroke, chronic lung disease, and cancer (Semenza et al., 2000).

## 4 HIFs in the microenvironment of BC

TME is a complex network composed of different cell types, signaling molecules, and extracellular matrix components, which together coordinate tumor progression (Catalano et al., 2013). The cellular components of the TME include cancer cells, surrounding immune cells and endothelial cells, cancer associated fibroblasts (CAFs), etc. (Spill et al., 2016; Del Prete et al., 2017). Similar to most solid tumors, hypoxia is an inherent property of the BC TME. HIF-1, as the driver of hypoxia, plays a key role in the activation of CAFs and it promotes persistent chronic inflammation in the TME (Whitaker-Menezes et al., 2011; Martinez-Outschoorn et al., 2014; Mao et al., 2021). In addition, immune evasion is considered to be one of the main strategies for tumor survival in the TME. HIF-1 signaling suppresses the immune system in the hypoxic TME, allowing cancer cells to evade immune responses by triggering the expression of immunosuppressive molecules (Barsoum et al., 2014; Semenza, 2014; Schito and Semenza, 2016; Jiang et al., 2019; You et al., 2021). Activation of the HIF signaling pathway maintains oxygen homeostasis by mediating the expression of multiple genes involved in regulating many critical functions of cells, including growth, metastasis, drug resistance, and maintenance of stemness (Bao et al., 2012; Semenza, 2017; Chen et al., 2020).

### 4.1 The association of HIFs and angiogenesis in BC

When in the initial stage of tumor growth (tumor volume <0.5 mm), tumor obtains nutrients and oxygen by diffusion, when tumor masses grow larger than 0.5 mm, nutrients obtained by diffusion are insufficient to sustain tumor growth, and new vasculature is formed to maintain the growth state (Hanahan and Weinberg, 2000). Activation of this “angiogenic switch” will form a new vasculature, which is inevitable for the growth and metastasis of malignant tumors (Hanahan and Folkman, 1996). Compared to normal tissue, tumor vascular distribution results in abnormal vascular distribution (dilated, tortuous, disorganized) and dysfunction (hyper penetration, edema) (Rapisarda and Melillo, 2012; Viallard and Larrivee, 2017). And tumor angiogenesis perfusion is reduced, which in turn exacerbates the hypoxic environment and maintains HIF-1α stability (Rey et al., 2017). Angiogenesis is also known as basic condition for tumor progression, proliferation and metastatic spread. HIFs is an important hub for regulating angiogenesis (Hashimoto and Shibasaki, 2015; Olejarz et al., 2020). BC angiogenesis can be activated by HIFs-mediated downstream pathways, primarily vascular endothelial growth factor (VEGF) (Darbeheshti et al., 2021). VEGF as one of the key downstream targets of HIFs pathway belongs to the endothelial growth factor family and plays a central role in angiogenesis through its effects on endothelial cell migration, proliferation, permeability and survival (Semenza, 2000; Schoppmann et al., 2006; Kallergi et al., 2009; Ahluwalia and Tarnawski, 2012; Saponaro et al., 2013). A study showed that RAB11B-AS1, a long noncoding RNA, enhances the expression of VEGFA and ANGPTL4 in hypoxic BC cells in a HIF2α-dependent manner, leading to tumor angiogenesis and metastasis (Niu et al., 2020). Research evidence has also shown that hypoxia could induce the HIF-1α/G-protein estrogen receptor (GPER) in CAFs, which regulates VEGF and finally elicit hypoxia-dependent tumor angiogenesis (De Francesco et al., 2013). It has also been shown by Kallergi et al. (2009) that HIF-1α co-express with VEGF in patients with metastatic breast cancer. The direct link between HIF-1α and VEGF suggests that HIF-1α has a profound role in angiogenesis, Anti-angiogenic therapies such as VEGF inhibitor may cause drug resistance by increasing intratumoral hypoxia and upregulating HIF-1α. Clinical trials have been designed to test the efficacy of bevacizumab combined with HIF-1α to conquer drug resistance (Falchook et al., 2014; Jeong et al., 2014).

### 4.2 The association of HIFs and glycolysis in BC

Increasing research evidence suggest that cancer is not only a genetic disease but also a metabolic disease, in which glycolysis is an important player. It has long been recognized that although there are adequate oxygen levels in the TME, the metabolic demands of cancer cells are shifted from aerobic respiration to the uptake of glycolytic glucose, the reprogram known as the Warburg effect. Another study also showed that HIF-1α may drive glycolysis independent of hypoxia TME, the targets of HIF-1in the glycolytic pathway include hexokinase 2 (HK2), lactate dehydrogenase A (LDHA) and glucose transporter 1 (GLUT1, also known as solute carrier family A1, SLC2A1), and accelerate the process of glycolysis by downregulating the expression of enzymes of the tricarboxylic acid cycle, the factors that contribute to this situation may be pyruvate kinase isoform M2 (PKM2) physically interacts with HIF-1 and stimulates HIF-1 activity, but not pyruvate kinase isoform M1 (PKM1) (Christofk et al., 2008; Luo et al., 2011). When cancer cell metabolism is shifted to aerobic glycolysis, pyruvate is replaced by lactate and released into the TME, creating an immunosuppressive environment that promotes tumor cell growth, metastasis and invasion (Liberti and Locasale, 2016; El-Sahli and Wang, 2020).

Hexokinase 2(HK2) is an enzyme that catalyzes the phosphorylation of hexose, it is the first and the rate-limiting enzyme of the glycolytic pathway. CircRNF20 is a 499 bp circular RNA derived from RNF20 Gene that can promote tumor progression *via* miR-487a/HIF-1α/HK2 in BC (Cao et al., 2020). O-linked-N-acetylglucosaminylation (O-GlcNAcylation) is a type of glycosylation, which regulates glycolysis *via* HIF-1α/GLUT1 signal pathway in BC cells (Ferrer et al., 2014). Circular RNA circRBM33 inhibits the expression of downstream glycolysis-related proteins (HK2, GLUT1) through the miR-542-3p/HIF-1α axis, thereby preventing glycolysis and promoting BC cell apoptosis (Jiang et al., 2022). However, other studies have shown that Pyruvate dehydrogenase kinase 1 (PDK1) is a key switch of tricarboxylic acid (TCA) cycle in mitochondria. Signal-induced proliferation-associated 1 (SIPA1), a member of Rap1GAP family, promotes aerobic glycolysis by regulating the SIPA1/HIF-2α/PDK1 axis, leading to tumor invasion and metastasis *in vivo* (Yao et al., 2021). Therefore, HIF-1 α plays an important role in glucose metabolism, and providing energy for cancer cells by controlling glucose metabolism may be another promising pathway.

**FIGURE 3:**
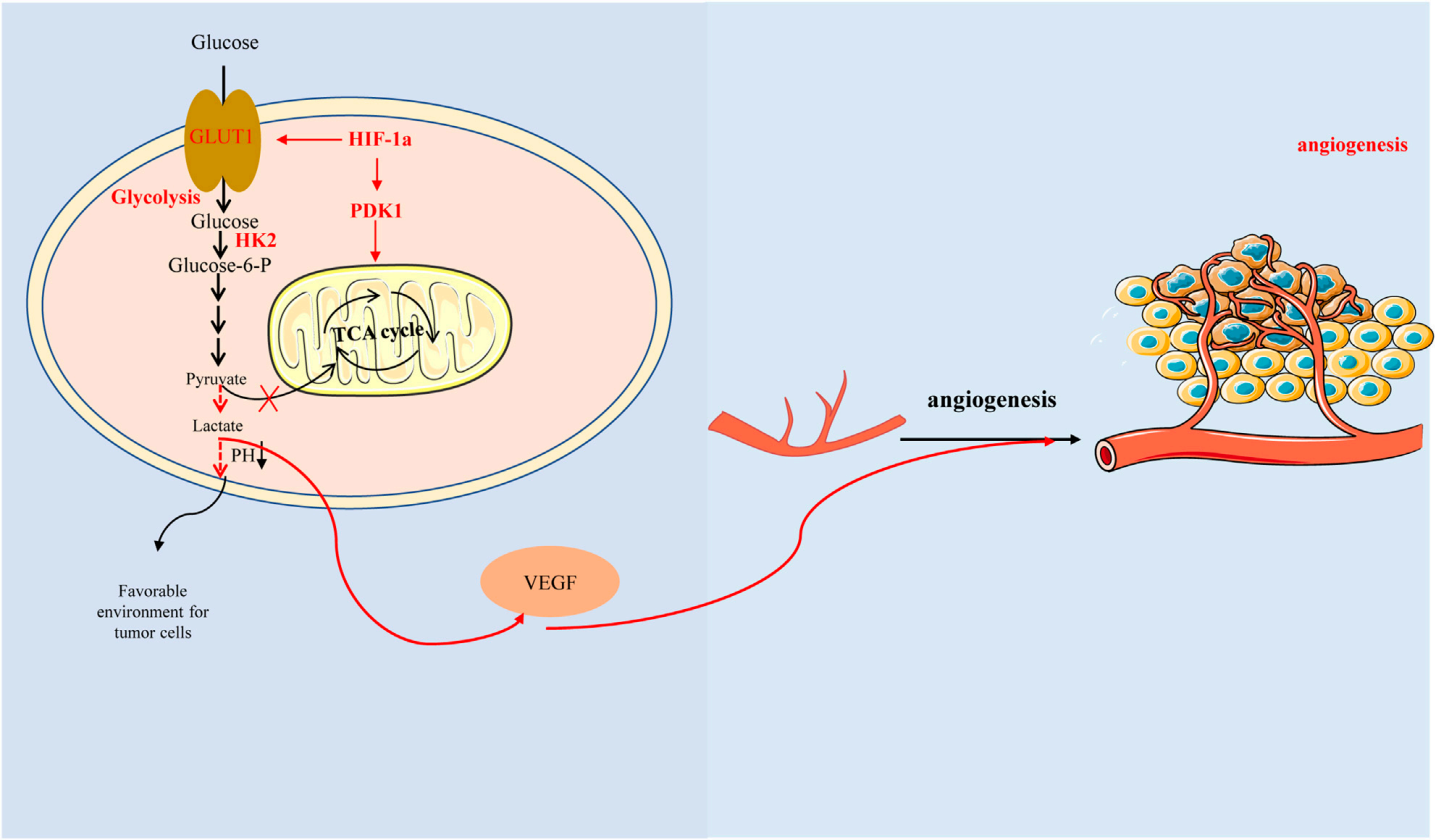
Hypoxia-inducible factor-1α promotes glycolysis by regulating key enzymes in the process of glycolysis, which produces lactic acid acidification microenvironment and affects glucose metabolism, thus promoting vascular endothelial growth factor expression and angiogenesis. In addition, HIF-1 α can also directly regulate tricarboxylic acid cycle and affect glucose metabolism with glycolysis-independent method.

Interestingly, here is a view that the ‘glycolytic switch’ occurs before the angiogenesis. Glycolysis could induce HIF-1α accumulation leading to high expression of VEGF (Figure 3) (Zare et al., 2021). Studies have reported that aerobic glycolysis can induce angiogenesis by producing lactate to acidify the extracellular environment and promote VEGF expression (Shi et al., 2001; Jung et al., 2011). Another study also suggested that lactate and pyruvate, the end products of glycolysis, regulate VEGF expression by increasing HIF-1α accumulation (Lu et al., 2002).

### 4.3 The association of HIFs with EMT and metastasis in BC

**FIGURE 4:**
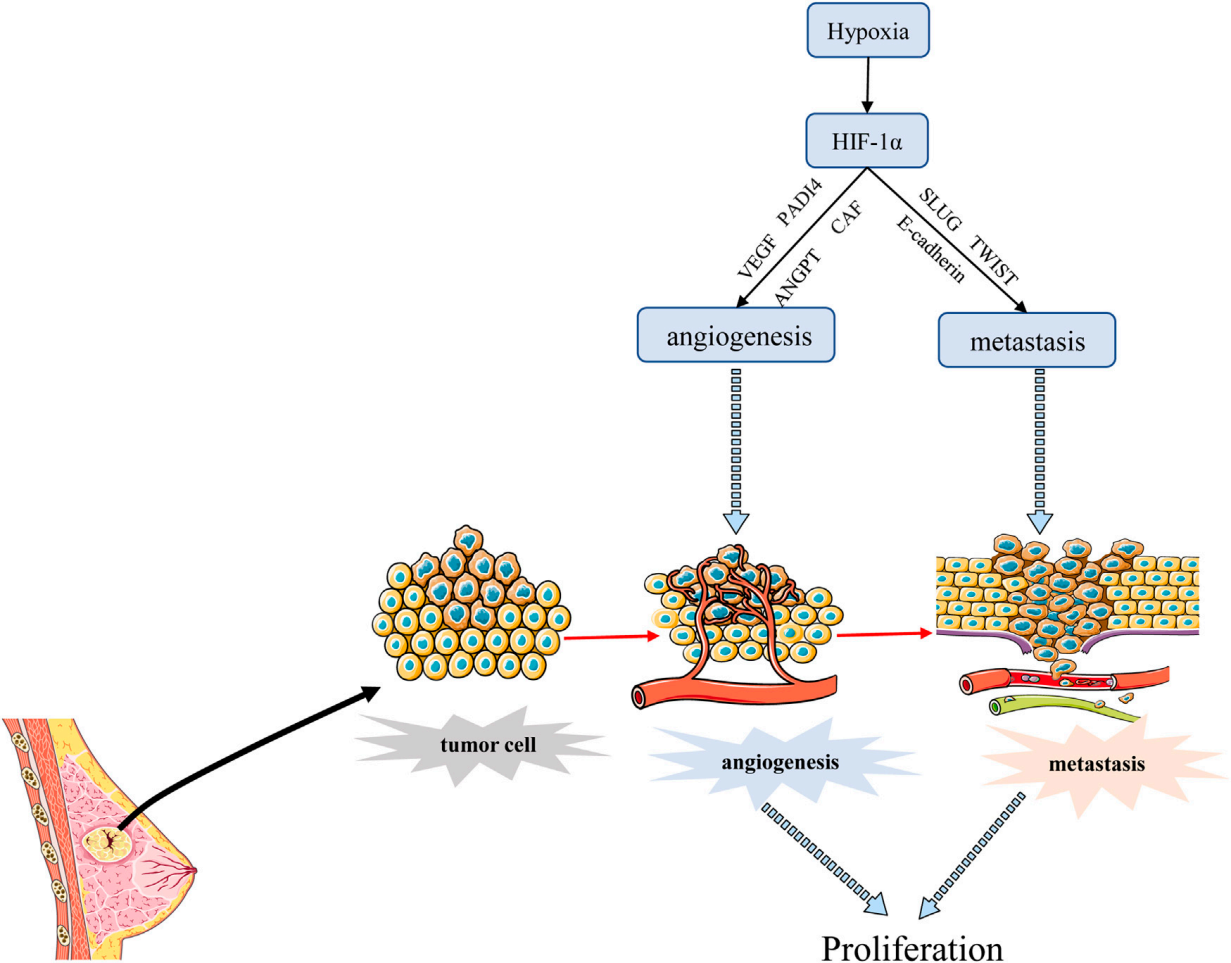
HIF-1α regulates the angiogenesis and metastasis of BC. HIF-1α promotes angiogenesis mainly by stimulating VEGF transcription, and promotes BC metastasis through EMT process. Angiogenesis can also promote transfer to normal tissues.

Tumor metastasis is a process by which cancer cells spread from the initial site of primary tumor growth to distant organs, where they survive, proliferate and form secondary tumors. EMT is an important part of tumor metastasis. It is the process of transformation from epithelial cells to cells with a mesenchymal phenotype through a specific program (Lee et al., 2006; Lee et al., 2011). Cancer cells that undergo EMT have strong invasive capacity and are resistance to apoptosis (Suarez-Carmona et al., 2017). Hypoxia is often an environmental feature of EMT, and activated HIF-1α induces cancer EMT through multiple molecules and pathways, including inflammatory cytokines, epigenetic regulators, and transcription factors (Bao et al., 2012). One study has shown that hypoxia induces HIF-1α expression, which induces the expression and activity of major transcription factors including TWIST, Snail, Slug, SIP1, STAT3, and ZEB, leading to the suppression of E-cadherin and induction of vimentin in BC cells. Inhibition of HIF-1α significantly enhanced the expression of E-cadherin (Zhou et al., 2016). Another report also revealed that hypoxia promoted the expression of Slug and Snail and decreased E-cadherin during HIF1-induced EMT through Notch pathway (Chen et al., 2010). He et al. (2020) demonstrated that hypoxia-induced HIF-1α regulated BC cells migration and EMT through the MiR-338-3p/ZEB2 axis. Moreover, research evidence showed that HIF-1α and integrin-linked kinase (ILK) formed a regulatory feedback loop which promoted EMT by modulating the expression of various EMT regulators/makers, including Snail, Zeb1, E-cadherin, and vimentin (Chou et al., 2015). Moon et al. (2021) found that MRPL52 acted as a transcriptional target of hypoxia-inducible factor (HIF-1α) and MRPL52 augmented epithelial-mesenchymal transition, migration and invasion of hypoxic BC cells by activating the ROS-Notch1-Snail signaling pathway. Angiopoietin-like protein ANGPTL4 is also a HIF-1α target that promotes lung metastasis when overexpressed in BC cells (Zhang et al., 2012). The above evidence suggested that HIF-1α acted as a crucial regulator of hypoxia induced EMT and metastasis through various mechanisms. In addition, angiogenesis is associated with metastasis because permeability and heterogeneous vascular systems contribute to the extravasation of tumor cells into normal tissues to escape the harsh hypoxia environment as shown in Figure 4. Metastasis is a major prognostic challenge for BC patients, and it may be a feasible way to inhibit BC metastasis by targeting HIF-1 α.

### 4.4 The association of HIFs and cancer stemness of BC

**FIGURE 5:**
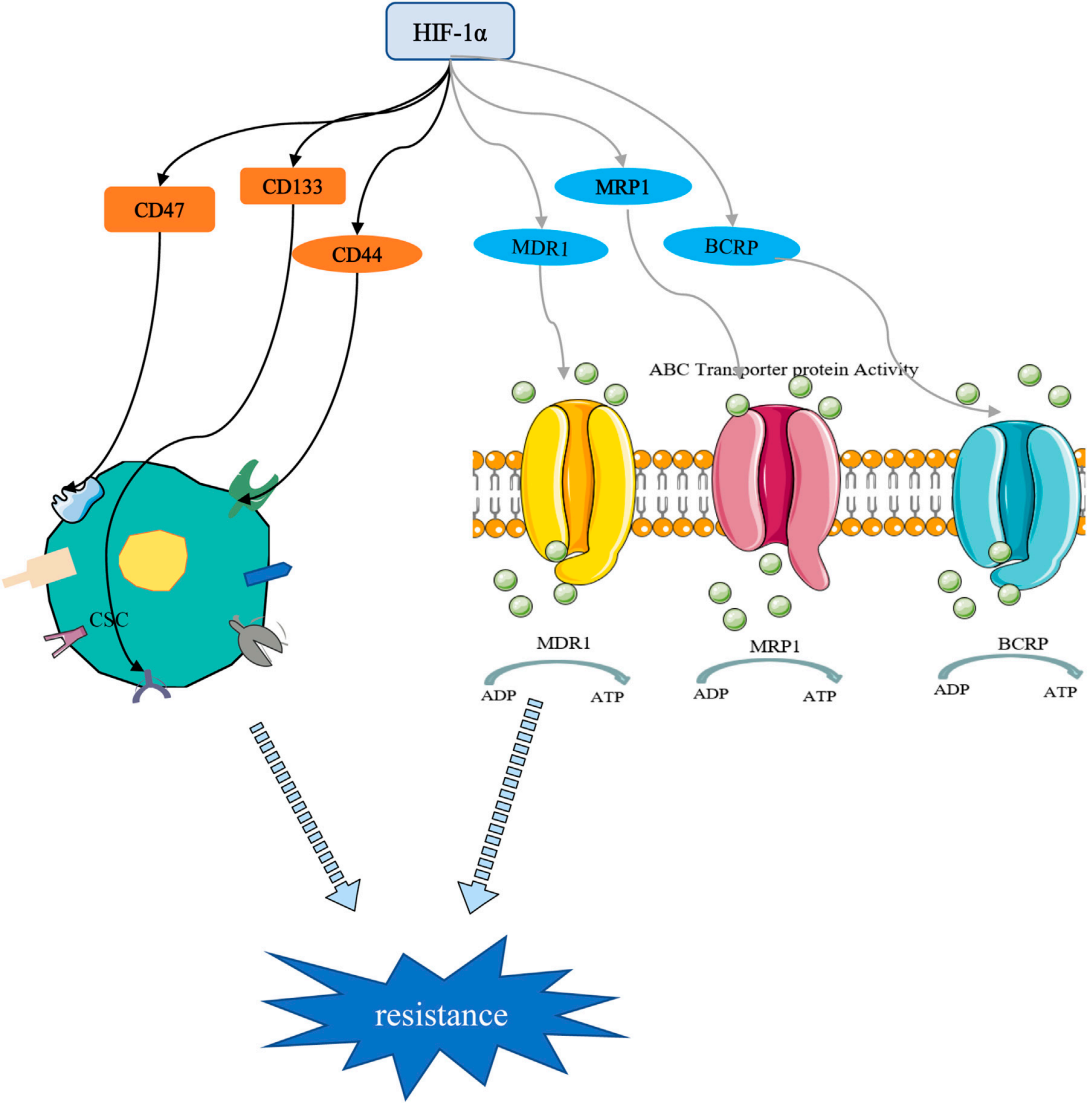
HIF-1α-mediated stemness and drug resistance. On the one hand, HIF-1α can induce drug resistance by regulating stem cell surface markers. On the other hand, HIF-1α promotes chemotherapy resistance through drug resistance-related proteins.

Cancer stem cells (CSCs) are a small subset of solid tumor cells with self-renewal and differentiation properties and tumorigenic potential and they spread to different parts of the body to form secondary tumors (Bai et al., 2018). Hypoxia may contribute to the formation of CSCs niches within tumors. Studies have confirmed that HIF targeting of cancer cell stemness-related genes may be a key inducer of stemness dynamics under pathological conditions. By increasing the expression of HIFs and enhancing the activity of HIFs, tumor cells acquire a stem phenotype and reach a higher degree of malignancy (Mohyeldin et al., 2010; Mathieu et al., 2011; Conley et al., 2012). Study has shown that the percentage of BC stem cells (BCSCs) is increased in a HIF-1 -dependent manner (Xiang et al., 2014). CD47 is a ubiquitously expressed cell surface glycoprotein belonging to the immunoglobulin superfamily, which is closely related to the self-renewal, tumorigenesis and chemotherapy resistance of BCSCs. When BC cells are in a hypoxic environment, HIF induces the CD47 expression to promote breast CSC phenotype (Zhang et al., 2015). As a widely distributed transmembrane glycoprotein, CD44 is one of the important markers of CSCs. It has been reported that down-regulation of HIF-2α expression can reduce the stemness of BC cells through the CD44/PI3K/AKT/mTOR signaling pathway (Zhang et al., 2015). Moreover, HIF-1α can regulate BC cell stemness by regulating CD133+ stem cell population (Schwab et al., 2012). These studies highlight the important role of HIFs in the maintenance of BCSCs (Figure 5).

### 4.5 The association of HIFs and drug resistance in BC

Chemotherapy drugs are still the cornerstone of cancer treatment. Their killing effect on tumor cells is oxygen-dependent, and most of them kill cells by oxidizing free radicals and reactive oxygen species in the cells. Long-term or severe hypoxic conditions have been shown to promote resistance of tumor cells to chemotherapeutic drugs (Harris, 2002; Semenza, 2007). Increased drug resistance in hypoxic tumors has been reported both in cells and animal models. Resistance has been attributed to the upregulation of HIF-1, which was associated with poor overall survival (Campbell et al., 2019; de Heer et al., 2020). The transcription of numerous target genes can be activated by HIF-1, which promoted physiological changes associated with treatment resistance, including multidrug resistance 1 protein (MDR1), multidrug resistance-related protein 1 (MRP1), and BC resistance protein (BCRP) (Figure 5). Doublier et al. (2012) found that HIF-1 is activated and participates in the transcriptional activity of the MDR-1 gene, which promotes the resistance of MCF-7 cells to doxorubicin by regulating the MDR1/P-glycoprotein (P-gp, ABCB1) axis. MRP1 is an organic anion transporter. Study has shown that knockdown of HIF-1α attenuated cheomoresistance *via* affecting the expressions of apoptosis-related molecules such as Bax and Bcl-2 and drug transporters as P-gp and MRP1 (Wang et al., 2018). Besides, HIF-1-mediated chemoresistance is closely related to autophagy, apoptosis, stemness and glycolysis (Mimeault and Batra, 2013; Chen F et al., 2019; Li et al., 2020).

Collectively, these findings highlight the importance of HIFs in carcinogenesis and progression, which have prompted the scientific community to focus on the importance of HIF-1 and enable the discovery of new drugs that specifically inhibit HIF-1α or its target genes.

## 5 Therapeutic strategies targeting HIF-1α in BC

Here we summarize HIF-1α inhibitors that are in clinical trials, and various compounds that target HIF-1α or the HIF-1α pathway in basic research. Finally, we will introduce the latest drug delivery systems for HIF-1 α, which are designed to improve drug selectivity and ensure drug concentration.

### 5.1 HIF-1α inhibitors in clinical trials

We found some clinical studies on HIF-1α-related drug therapy for breast cancer as shown in Table 1. Unfortunately, they did not achieve some of the expected results. Three projects were completed in the second phase, but none of the projects successfully collected data on the response to HIF-1 α treatment, and two of them were terminated in advance. As for the reasons for early termination of the study, a small sample size and a small number of patients are the main causes, while serious adverse events caused by non-specific cytotoxicity are another possibility. As a result, more specific and safer preparations are needed for targeting HIF-1α in BC in clinic, which may take some time.

**TABLE 1:** HIF-1α related clinical studies in BC.

Drug	Status	Phase	Main Outcomes	Toxicity	NCT Number
Digoxin	Completed	Phase 2	There was not enough data to analyze HIF-1alpha expression because of the limited tumor samples	No over grade 2 adverse event related to digoxin occurred	NCT01763931
Vinorelbine	Completed	Phase 2	The study was terminated early	Elevated liver enzymes (grade 3) 22.2%, Febrile infection (grade 5) 11.1%	NCT03007992
Paclitaxel plus bevacizumab	Completed		There was no significant difference between HIF-1alpha polymorphism and longer PFS in patients treated with paclitaxel and bevacizumab		NCT01935102
Bevacizumab, docetaxel	Completed	Phase 2		The rate of serious adverse events is about 18.06% and the rate of other adverse events is 98.61% in total	NCT00559754
Propofol, Sevoflurane	Unknown	Not Applicable			NCT03005860

### 5.2 HIF-1α inhibitors under investigation

Since HIF-1α is closely related to the key processes in tumor progression and its expression is also associated with patient survival, it is not surprising that targeting HIF-1α has been extensively studied as possible therapeutic strategy against cancers. At present, there are no HIF-1α inhibitors approved by the FDA for BC treatment. The reported HIF-1α inhibitors for BC are still in basic research, so there is still an urgent need to discover novel HIF-1α inhibitors with sufficient potency, low toxicity, good druggability. HIF-1α inhibitors work by suppressing different processes in the HIF pathway: 1) HIF-1a protein accumulation 2) DNA binding 3) transcriptional activity 4) HIF-1a translation. Small molecule inhibitors targeting HIF-1α are shown in Table 2.

**TABLE 2:** HIF-1α inhibitors under investigation in BC.

Compound	Dose	HIF-1 activity IC_50_	Cell growth inhibition IC_50_	Model	Duration of treatment	Routes of administration	Type of study	Mechanism	Results	References
KC7F2	40 uM	15 uM	20 uM	MCF-7	8–72 h	—	*in vitro*	decrease HIF-1a protein accumulation	Inhibit cancer cell growth in a dose-independent manner	Narita et al. (2009)
LXY6090	0.4 nM-100uM	4.11 ± 0.4 nM	T47D: 245.7 ± 15.2 nM; MCF-7: 352.7 ± 14.2 nM; MX-1: 108.2 ± 2.1 nM	T47D, MCF-7, MX-1	16–96 h	—	*in vitro*	downregulate HIF-1a protein and mRNA level by promoting HIF-1a proteasome degradation	Inhibit breast cancer cells growth dose-dependently	Lai et al. (2016)
	25 mg/kg/d to 100 mg/kg/day	—	—	Mouse model (MX-1)	14 days	ip	*in vivo*	depress HIF-1a expression *in vivo*	Inhibit MX-1 cells subcutaneous xenograft tumors growth in a dose dependent manner	
Quercetin	10–100 uM	—	—	SkBr3	1–8 h	—	*in vitro*	inhibiting HIF-1a protein accumulation	Did not affect cancer cell activity	Lee and Lee, (2008)
Aryl Carboxamide Derivatives (30 m**)**	0.5–30 uM	0.32 uM	—	MDA-MB-231	24 h	—	*In vitro*	inhibit HIF-1a protein accumulation and promote its degradation	Suppress cancer cells angiogenesis activity dose-dependently, and inhibit cancer cell invasion and migration	Liu et al. (2019)
	15–30 uM/2 days	—	—	Mouse model (MDA-MB-231)	3 weeks	ig	*in vivo*	inhibit HIF-1a protein accumulation	Inhibit lung colonization of tumor cells without obvious body weight loss in a dose-depended manner	
LXY6006	0.1–1 uM	0.35 ± 0.11 nM	1.3–249.7 nM	T47D, MD-MBA-231, MX-1	4–5 days	—	*in vitro*	inhibit HIF-1a nuclear accumulation	Arrest cell cycle, and hold back cancer cells growth	Lang et al. (2014)
LXY6006	60 or 120 mg/kg/6 days per week	—	—	Mouse model (MX-1 or MX-1/Taxol)	13 days	ig	*in vivo*	—	Arrest both normal and taxol-resistant breast cancer xenograft growth with slight body weight loss	
Aminoflavone	0.06-1uM	—	—	MCF-7	16 h	—	*in vitro*	depress HIF-1a protein accumulation and decreases the rate of HIF-1α translation	Shows cytotoxic effect on breast cancer cells	Terzuoli et al. (2010)
Aminoflavone	60 mg/kg/day	—	—	Mouse model (MCF-7)	4 days	—	*in vivo*	AF inhibits HIF-1α expression	Inhibit cancer growth	
7-Hydroxyneolamellarin A	0.6–50 μmol/L	23.0 ± 2.6 μmol/L	—	MCF-7,4T1	4–36 h	—	*in vitro*	inhibit HIF-1a protein accumulation	Suppress the cellular migration, invasion and proliferation dose-dependently	Li et al. (2021)
7-Hydroxyneolamellarin A	15 mg/kg/2 days	—	—	Mouse model (4T1)	23 days	—	*in vivo*	inhibit HIF-1a protein accumulation	Inhibit HIF-1a and breast tumor growth with slightly body weight effect	Li et al. (2021)
DJ12	2.5–100 uM	3.6 uM	165–250 uM	MDA-468, ZR-75, MD435	16 h	—	*in vitro*	decrease HIF-1a transactivation and DNA binding	—	Jones and Harris, (2006)
Cardenolides	—	21.8–64.9 nM	30.5–68.8 nM	MCF-7	24 h	—	*in vitro*	inhibited HIF-1 transcriptional activity dose-dependently	cytotoxic effects on breast cancer cells	Parhira et al. (2016)
PX-478	—	—	—	Mouse model (MCF-7)	—	—	*in vivo*	suppresses HIF-1a levels	antitumor activity	Welsh et al. (2004)
Methylalpinumisoflavone	0.01–10 uM	0.6 μM	—	T47D, MDA-MB-231	24–48 h	—	*in vitro*	inhibits HIF-1 activation by blocking the induction of nuclear HIF-1α protein	Inhibit tumor angiogenesis *in vitro*, cell migration, and chemotaxis	Liu et al. (2009)

For example, based on Aryl Carboxamide Derivatives, 68 new aryl carboxamide compounds were synthesized and inhibitory effect was evaluated by dual luciferase-reporter assay. The results showed that compound 30 m was the most active inhibitor with the lowest cytotoxicity. It effectively attenuated hypoxia-induced HIF-1α protein accumulation in a dose-dependent manner, which was demonstrated by its inhibitory potency on capillary-like tube formation (Liu et al., 2019). In another study, cardenolides were isolated and purified from latex and giant fir fruit of Calotropis gigantea, a medicinal plant. These cardenolides inhibited HIF-1α transcriptional activity and exhibited potent cytotoxicity with a dose-dependent manner in MCF-7 cells, but minimal inhibitory effect on normal human breast cells (Parhira et al., 2016).

### 5.3 Small molecule compounds targeting HIF-1α related signaling pathway in BC

Using extracts from medicinal plants and chemically synthesized derivatives have become the current trend in drug development. Table 3 summarizes the small molecule compounds targeting HIF-1-related signaling pathways in BC, which mainly act on key genes regulated by HIF-1 including those involved in glycolysis, angiogenesis and metastasis.

**TABLE 3:** HIF-1 related signaling pathway inhibitors in BC.

Component	Dose	IC_50_	Model	Duration of treatment	Routes of treatment	Type of study	Mechanism	Results	References
Honokiol	0–40 uM	—	MCF-7, MDA-MB-231	3–24 h	—	*in vitro*	downregulated HIF-1α protein expression	Inhibited cell proliferation and clonogenicity, as well as induced apoptosis of cancer cells	Yi et al. (2022)
	25 mg/kg/day	—	Mouse model (MCF-7)	4 weeks	ip	*in vivo*	decrease HIF-1α protein level	Suppressed tumor growth and HIF-1α-mediated glycolysis	
Sinomenine	0.75 mM	—	stem-like side population (SP) cells gained from MDA-MB-231	24 h	—	*in vitro*	downregulating HIF-1α	Inhibit the migration and vasculogenic mimicry, and hold back epithelial-mesenchymal transition process	Song et al. (2022)
Polydatin (PD) combined with 2-deoxy-D-glucose (2-DG)	PD 100 μmol/L, 2-DG 5 mmol/L (4T1) or 10 mmol/L (MCF-7)	PD: 66.56uM(4T1)/103.1 uM (<CF-7), 2-DG: 5.53 mM(4T1)/8.67 mM (MCF-7). (24 h)	MCF-7 and 4T1	0–72 h	—	*in vitro*	inhibit HIF-1alpha/HK2 to suppress glycolytic metabolism	Induced cell apoptosis and inhibited cancer cells proliferation, migration and invasion	Zhang et al. (2019)
Polydatin (PD) combined with 2-deoxy-D-glucose (2-DG)	PD (100 mg/kg every other day), 2-DG (100 mg/kg ip every other day)	—	Mouse model (4T1)	3 weeks	ip	*in vivo*	anti-proliferative and anti-angiogenic activity, promoted apoptosis	Inhibit cancer growth *in vivo*	Zhang et al. (2019)
bishonokiol A	2.5–10 uM	—	MCF-7, MDA-MB-231	24–48 h	—	*in vitro*	hold back HIF-1a expression and its protein synthesis	Inhibit cancer cell invasion and migration	Li H. M et al. (2019)
	100 mg/kg/3 days	—	Mouse model (MDA-MB-231)	16 days	ip	*in vivo*	—	Antitumor activity and low toxicity	
Alkaloid derivative ION-31a	0–75 uM	—	MDA-MB231, 4T1	24 h–48 h	—	*in vitro*	downregulate HIF-1α/VEGF signaling pathway	Inhibit cell migration, invasion, adhesion, and VEGF secretion	Ni et al. (2021)
Alkaloid derivative ION-31a	25–100 mg/kg	—	Mouse model (4T1)	26 days	ig	*in vivo*	—	Depress tumor growth and metastasis with slightly bodyweight change	
HS-1793	0–50 uM	MCF-7: 26.3 ± 3.2; MDA-MB-231: 48.2 ± 4.2 uM	MCF-7 and MDA-MB-231	24 h	—	*in vitro*	downregulate HIF-1a protein level and its target gene VEGF expression	inhibit cancer cells proliferation, and decrease the angiogenesis	Kim et al. (2017)
	0–20 mg/kg/twice a week	—	Mouse model (MDA-MB-231)	4 weeks	ip	*in vivo*	downregulate HIF-1a protein level	Inhibit tumor growth, and suppress microvessel formation	
Salinomycin	0–30 uM	—	MCF-7, T47D, MDA-MB-231, MDA-MB-468, 4T1	12–24 h	—	*in vitro*	decreased the HIF-1α transcription factor DNA binding activity	Inhibit cell proliferation, invasion, and migration	Dewangan et al. (2019)
	5–10 mg/kg/3 days a week	—	Mouse model (4T1)	3 weeks	ip	*in vivo*	inhibited hypoxia-induced HIF-1α/VEGF signaling axis	inhibits breast cancer growth and tumor angiogenesis	
HS-146	—	—	MCF-7	—	—	*in vitro*	depress hypoxia-induced HIF-1α/VEGF signaling axis	Inhibit cancer cell proliferation, migration and invasion in a dose-dependent manner	Kim et al. (2020)
Baicalein	0–25 uM	—	T-47D, BT-474 and ZR-75–1	24–72 h	—	*in vitro*	inhibit HIF-1α–mediated aerobic glycolysis and mitochondrial dysfunction	—	Chen et al. (2021)
	30 mg/kg/3 days	—	Mouse model (MCF-7TR)	30 days		*in vivo*	inhibit HIF-1α–mediated aerobic glycolysis and mitochondrial dysfunction	Baicalein increases the inhibitory effects of TAM on the growth of MCF-7TR cells *in vivo*	
Chiral ionone alkaloid derivatives	0–30 uM	0.035 μM ± 0.004	MDA-MB-231	0–24 h	—	*in vitro*	inhibit HIF-1α/VEGF/VEGFR2/Akt pathway	Depress cancer cell migration, adhesion, migration and invasion	Liu J. J et al. (2021)
Cardamonin	—	24.458–52.885 uM	MDA-MB-231	24–72 h	—	*in vitro*	inhibit HIF-1a expression on mRNA and protein level	Inhibit cancer cell viability and promotes apoptosis	Jin et al. (2019)
	3 mg/kg/day	—	Mouse model (MDA-MB-231)	4 weeks	ip	*in vivo*	suppress HIF-1α/PDHK1 axis by inhibit the mTOR/p70S6K pathway	Inhibit tumor growth	
AT-533	0–75 uM	—	MDA-MB-231, MCF-7	12–72 h	—	*in vitro*	downregulate HIF-1α/VEGF signaling pathway	Inhibit breast cancer cells viability	Zhang et al. (2020)
	10 mg/kg/2 days	—	Mouse model (MDA-MB-231)	12 days	ip	*in vivo*	block the HIF-1α/VEGF/VEGFR-2-mediated signaling pathway	Inhibit growth of breast cancer xenografts *in vivo*	
Rhaponticin	0–100 uM	—	MDA-MB231	48 h	—	*in vitro*	decreased HIF-1α accumulation and HIF-1α nuclear expression	suppress cancer cells colony formation, migration, invasion and angiogenesis	Kim and Ma, (2018)

For instance, Honokiol (HNK), a natural compound, inhibited the glycolysis of BC cells and indirectly blocked tumor growth by targeting HIF-1α/GLUT1/PDK1/HK2 pathway (Yi et al., 2022). In another report, the researchers synthesized ionone alkaloid derivatives and identified the compound ION-31a with anti-metastatic activity of BC. Although ION-31a is a heat shock protein 90 (HSP90) inhibitor, it significantly inhibits BC metastasis and angiogenesis by HSP90/HIF-1α/VEGF/VEGFR2 signaling pathway (Ni et al., 2021).

### 5.4 Drug delivery system targeting HIF-1α in BC

Here we highlight the HIF related drug delivery systems, including liposomes NPs, polymers NPs, metal-based NPs or carbon-based NPs. A few experimental drug delivery systems targeting HIF-1α are contained in Table 4.

**TABLE 4:** Drug delivery systems for targeting HIF-1α.

Carrier and feature	Pharmaceutical ingredients	Cell line	References
HPDA	BEZ235	4T1	Liu et al. (2022)
PLGA-NP	Curcumin	MDA-MB231	Khan et al. (2018)
FA-BSA-MnO2	DOX/siRNA	MCF-7	Du et al. (2019)
PVCL-PVA-PEG	Betulinic acid	MDA-MB-231	Qi et al. (2021)
SPION-TMC-ChT-TAT-H NPs	siRNA	4T1	Budi et al. (2021)
ONB	Dox	MDA-MB-231	Khan et al. (2019)
Carbon nanoparticles	docetaxel	Walker256	Liu W et al. (2021)
Liposomal	echinomycin	MCF-7/SUM-159/MDA-MB-231	Bailey et al. (2020)
RBCm	Sal/ICG	4T1	Pan et al. (2022)

#### 5.4.1 Liposomal NPs

Liposome NPs (LNPs), a kind of spherical vesicles with a size of several hundred nanometers, can encapsulate drug molecules with vesicles from phospholipid bilayer membranes. Liposomes have several additional advantages as nanocarriers for drug delivery applications. Liposomes protect the loaded drug from degradation, reduce the rate of drug release and the toxicity of drugs due to non-target distribution (Allen and Cleland, 1980; Senior and Gregoriadis, 1982; Bobo et al., 2016). LNPs are also potential delivery carriers for hydrophilic agents by encapsulating them in the inner core.

Acriflavine (ACF) is a kind of drug that inhibits Hypoxia-inducible factor (HIF) pathway and exerts cytotoxicity. One study demonstrated that compared with free drug, liposome encapsulated ACF showed similar cytotoxicity in 4T1 cells and decreased HIF activity *in vitro*. Compared with free ACF, liposome encapsulated ACF (ACF dose of 5 mg/kg) showed higher anti-tumor efficacy in an orthotopic model of murine breast cancer (4T1 cells) *in vivo* (Montigaud et al., 2018). R8 polypeptide, a small molecule cell-penetrating peptide, can carry macromolecular substances into cells and increase active targeting of drugs (Kang et al., 2017), R8GD modified daunorubicin liposomes plus R8GD modified emodin liposomes had small and uniform particle size and high drug encapsulation rate, which allowed the chemotherapeutic drug to selectively accumulate at tumor site. VM channels and metastasis are effectively inhibited compared with free drug in MDA-MB-435 cell, which may be related to down-regulation of metastasis related proteins, including HIF-1 α (Fu et al., 2020). In another report, researchers developed a new targeted liposome mitochondrial tropical material D-a-tocopheryl polyethylene glycol 1000 succinate-triphenylphosphine conjugate (TPGS1000-TPP) to encapsulate sunitinib and vinorelbine respectively. Targeted drug liposomes are accumulated in the mitochondria of invasive breast cancer cells or VM channel forming cancer cells. It can induce acute cytotoxic injury and apoptosis and down-regulated VM channel forming indicators (MMP-9, EphA2, VE cadherin, FAK and HIF-1 α) (Shi et al., 2015). Ying Li et al. found that a cationic liposome technology can rapidly release mesenchymal-epithelial transition to enhance the cytotoxicity of doxorubicin by reduce hypoxia stress *in vivo* and inhibit HIF-1α expression *in vitro* (Li Y et al., 2019). LNPs has been identified as an effective delivery model for peptide and siRNA-based BC gene therapy. Encapsulation of these peptides and siRNAs with LNPs prevents their degradation in the vasculature environment and allows targeted delivery by using target ligands. Emine Ş Alva et al. showed that chitosan coated liposome targeted HIF-1α siRNA and VEGF siRNA can improve the efficiency of gene silencing. The siRNA-based therapy of chitosan coated liposomes may have potential in cancer treatment (Hortobagyi et al., 2001; Salva et al., 2015). Ju et al. (2014) also reported liposomes modified with PTD (HIV-1) peptide, which contains epirubicin and celecoxib, to target vasculogenic mimicry channels in invasive breast cancer. In the study of Khan et al. (2019), phospholipids, as a component of liposomes, are also an easily synthesized, biocompatible and biodegradable carrier. They used phospholipids as shells to encapsulate doxorubicin and synthesize doxorubicin loaded oxygen nanobubbles (Dox/ONB), compared with free drugs. Dox/ONB significantly inhibited HIF-1α activity and increased ROS production to enhance the antitumor effect of doxorubicin under hypoxia in breast cancer cells.

Therefore, LNPs are very popular as nano-carriers of biodegradable drugs. These drugs can be encapsulated and protected until they reach the target cells, which is particularly important for peptides and siRNAs. In addition, in order to achieve better biocompatibility, LNP is usually coated with polymer, which increases the liposome size, and the drug release process may be affected by opening the phospholipid bilayer.

#### 5.4.2 Metal-based NPs

Metal nanomaterials, also known as metal oxide nanomaterials, contain the core of magnetic and optical properties and the shell of the machine surface coating, which can make drugs gather in the local part of the body under the action of external magnetic field. Superparamagnetic iron oxide NP(SPION-NP) is a kind of magnetic nanomaterials. Researchers used SPION-NPs coated with thiolated chitosan (ChT) and trimethyl chitosan (TMC) and functionalized with hyaluronate (H) and TAT peptide for delivery of siRNA molecules against STAT3 and HIF-1α to cancer cells both *in vivo* and *in vitro*. The results indicated that tumor cell transfection with siRNA-encapsulated NPs robustly inhibited proliferation and migration and induced apoptosis in breast cancer cells (Budi et al., 2021). Similarly, researchers utilized superparamagnetic iron oxide-based NPs (SPIONs) combined with chitosan lactate (CL) and folic acid (FA) nanoparticles (NPs) loaded with TIGIT-siRNA and HIF-1α-siRNA for suppressing TIGIT and HIF-1α in tumor cells in another study. Results showed that cancer cells treated with TIGIT and HIF-1α siRNA-loaded SPIONs-CL-FA NPs strongly suppressed the TIGIT and HIF-1α expression and cancer angiogenesis (Fathi et al., 2021). At present, there are only a few studies on metal nanocarriers targeting HIF-1α in breast cancer and SPION has certain toxicity. More optimized metal nanocarriers may be developed in the future.

#### 5.4.3 Polymer-based NPs

Polymer-based NPs (PNPs) have been extensively studied as drug delivery vehicles. PNPs are usually prepared by combining a copolymer with another polymer matrix. Polymer-based NPs can be synthesized from native polymers, such as hyaluronic acid, chitosan (Agnihotri et al., 2004; Choi et al., 2010), as well as synthetic polymers such as polyglycolic acid (PGA), poly (lactate-coethylene glycol) (PLGA). Polylactic acid (PLA), polyllactide-coethyl ester (PLGA) and chitosan are the most typical biodegradable and biocompatible polymers. Anticancer drugs can be incorporated into the surface of PNP by surface adsorption, chemical coupling or encapsulation. Curcumin is a NF-κβ inhibitor. A study reported that researchers fabricated biodegradable poly (lactic-co-glycolic acid) PLGA nanoparticles (NP) loaded with curcumin (cur-PLGA-NP). These nanoparticles effectively facilitated the targeting of curcumin by delivering to the tumor site in the form of nanoparticles in the hypoxic micro-environment. Compared with free curcumin, the nano-formulation group has increased solubility and anti-tumor activity, which can effectively improve the tumor hypoxic microenvironment and block the occurrence and development of tumors by suppressing HIF-1α (Khan et al., 2018). Botulinic acid (3β-Hydroxy-20 (29)-lupaene-28-oic acid, BA) is a kind of pentacyclic triterpenoids with various biological activities such as antitumor, antiviral, anti-inflammatory and antioxidant. Due to poor solubility and low bioavailability, it cannot be used to effectively treat BC. In order to improve the antitumor activity of BA, researchers prepared polyvinyl caprolactam-polyvinyl acetate-polyethylene glycol (PVCL-PVA-PEG) grafts Copolymer (Soluplus) encapsulated BA micelles, which inhibit the angiogenesis of BC cells by suppressing the HIF-1/VEGF/FAK signaling pathway (Qi et al., 2021). Similarly, Betulinic acid (3β-Hydroxy-20 (29)-lupaene-28-oic acid, BA) has excellent anti-cancer activity but low bioavailability for poor solubility. A polyvinyl caprolactam–polyvinyl acetate–polyethylene glycol (PVCL–PVA–PEG) graft copolymer (Soluplus) encapsulated BA micelle (Soluplus-BA) was fabricated and results showed that Soluplus-BA micelles increased the inhibitory effect of BA on the angiogenesis by regulating the HIF-1/VEGF-FAK signaling pathway in breast cancer MDA-MB-231 cells (Qi et al., 2021). Recently a photodynamic therapy based on conjugated PNPs for BC has been reported (Liu et al., 2022). Ying Zhang et al. (2022) synthesized photochemical-responsive nanoparticle by incorporating DOX, curcumin (CUR), and perfluorooctyl bromide (PFOB) into poly (lactic-co-glycolic acid) (PLGA) *via* double emulsification (DOX-CUR-PFOB-PLGA). The synthesized composite nanoparticles with good ultrasound imaging induced MCF-7 cells apoptosis by downregulating AKT/HIF-1α signaling pathway. A drug delivery nanoplatform equipped with dual PI3K/mTOR inhibitor Dactolisib (NVP-BEZ235, BEZ235) and CAIX inhibitor 4-(2-aminoethyl) benzene sulfonamide (ABS) was designed to form HPDA-ABS/PEG-BEZ235/Ce6 (H-APBC) nanoparticles. The study showed that the H-APBC could produce ROS upon light irradiation and release of BEZ235 from H-APBC in acid microenvironment could mitigate PI3K/mTOR signal and resist HIF-1α-dependent tumor hypoxia adaptation (Liu et al., 2022). Photodynamic therapy (PDT) has become an emerging area of modern medicine. Studies have shown that the synergistic effect of PDT could enhance the effectiveness and reduce the limitations of the original treatment modality (Chen L et al., 2019; Xie et al., 2020).

#### 5.4.4. Carbon-based NPs

Carbon nanotubes (CNTS) have a cylindrical shape with a long, hollow structure and a wall formed of graphene sheets. Carbon nanotubes have the advantages of thermal conductivity, optical and electrical properties. In addition, as nanocorbs. CNTS can act as excellent optical absorbers in near-infrared (NIR) light due to their tunable surfaces and unique thermal properties. Researchers designed a novel targeted multifunctional nanoplatform, which refers to docetaxel (DOC) and perfluorohexane (PFH) loaded onto carbon nanoparticles (CNs), and combined them with anti-HIF-1α antibody-modified PLGA nanoparticles (HPDC NPs) to achieve dual US/PA imaging-guided and laser-triggered *in situ* DOC release. HPDC NPs efficiently deliver CNs and DOC into lymph nodes to achieve their targeting behavior and the nanoparticles can be destroyed under NIR-I laser irradiation and subsequently release DOC molecules. This study not only provides targeted chemotherapy-hyperthermia synergistic therapy by laser-triggered, highly efficient *in situ* chemotherapeutic nano systems, but also represent a nano-delivery route that avoids additional damage from drug entry into the bloodstream (Liu W et al., 2021). Compared with metal-based NPs, carbon based NPs can be considered as a more promising DDS for cancer treatment and diagnosis. However, the preparation of carbon nanotubes is complex and there is a challenge in poor solubility and biodegradability of CNT (Mehra et al., 2008).

## 6 Conclusions and future perspectives

The recent in-depth refinement and diversify of treatments modalities for BC have led to significant control of tumors as well as improved patient prognosis. However, these treatments are considered as only temporary control of metastasis and primary tumors, and most patients often face recurrence and metastasis after treatment. HIF-1 may promote the development of BC through a series of downstream pathways, and its overexpression is related to tumor progression and BC mortality. For this reason, HIF-1 may be a potential therapeutic target in BC. However, HIF-1 inhibitors are very rare in clinic. Although more and more HIF inhibitors have been found, they are still inadequate as for drug selectivity and specificity. In addition, HIF has complex interactions among multiple pathways, which makes the clinical application of HIF inhibitors more challenging. Therefore, at this stage, we believe that it is a prerequisite to develop specific HIF-1 inhibitors and further clarify the regulatory pathway of HIF. In addition, the upstream governor of HIF-1 is also an attractive strategy, and a deeper understanding of the regulatory mechanism of the upstream regulator of HIF-1 will help us to start new therapeutic interventions. On the other hand, improving targeting specificity, overcoming solubility and reducing drug toxicity have attracted widespread attention on the drug delivery system based on nano-carriers, while only a few drugs based on nano-carriers are used in preclinical research stage. The toxicity of nano-carriers to the body and the metabolism of drugs loaded on nano-carriers is a complex topic. It may be necessary to find non-toxic nanoscale carriers and to test the metabolic changes of nanomaterials in *vivo* model. With the progress of nano-biotechnology and the development of cancer treatment, we believe that the difficulty of nano-carrier in clinical treatment of BC will be broken through, and more drugs based on nano-materials will benefit BC patients. Overall, targeting hypoxia is a very promising way for cancer therapy but its real fulfillment requires time and great efforts.

## Abbreviations

HIFs, hypoxia-inducible factors; DDS, newly discovered drug delivery systems; TNBC, triple-negative BC; BC, breast cancer; RBCm, anti-LDLR engineered red blood cell membranes; Sal, salidroside; ICG, indocyanine green; TME, the tumor microenvironment; PHD, prolyl hydroxylase domain protein; HREs, hypoxia response elements; BHLH, basic helix-loop-helix; PAS, Per-Arnt-Sim; ECM, extracellular matrix; CAF, cancer-associated fibroblasts; EMT, epithelial-mesenchymal transition; VEGF, vascular endothelial growth factor; VHL, VonHippel-Lindau; TAM, tumor-associated macrophages; TCA, enzymes of the tricarboxylic acid cycle; GLUT1, glucose transporter 1; HK2, Hexokinase 2; CSC, Cancer Stem Cell; BCSC, BC Stem Cell; Snail, zinc finger transcription factor; NP, nanoparticle; DOX, doxorubicin; ACF, acriflavine.
